# Incidence of second primary malignancies in women with different stages of breast cancer

**DOI:** 10.3389/fonc.2022.1047684

**Published:** 2023-01-09

**Authors:** Cheng-Yao Lin, Sheng-Yen Hsiao, Wen-Tsung Huang, Chao-Jung Tsao, Chung-Han Ho, Shih-Bin Su, How-Ran Guo

**Affiliations:** ^1^ Division of Hematology-Oncology, Department of Internal Medicine, Chi Mei Medical Center, Tainan, Taiwan; ^2^ Department of Senior Welfare and Services, Southern Taiwan University of Science and Technology, Tainan, Taiwan; ^3^ Department of Environmental and Occupational Health, National Cheng Kung University, Tainan, Taiwan; ^4^ Department of Medical Research, Chi Mei Medical Center, Tainan, Taiwan; ^5^ Department of Hospital and Health Care Administration, Chia Nan University of Pharmacy and Science, Tainan, Taiwan; ^6^ Department of Medical Research, Chi Mei Medical Center, Liouying, Tainan, Taiwan; ^7^ Department of Occupational Medicine, Chi-Mei Medical Center, Tainan, Taiwan; ^8^ Department of Occupational and Environmental Medicine, National Cheng Kung University Hospital, Tainan, Taiwan

**Keywords:** second primary malignancies, breast cancer, chemotherapy, radiotherapy, cancer stage

## Abstract

**Introduction:**

Breast cancer (BC) is the most common cancer in women worldwide. Because of the extended survival of patients with BC, the occurrence of second primary malignancies (SPMs) after BC is an important issue.

**Methods:**

We identified female patients with BC in the Breast Cancer Health Database of Taiwan, which includes four cancer registry datasets between 2002 and 2014 from Taiwan Cancer Registry. We compared the incidence of SPM between patients who received chemotherapy and/or radiotherapy with those who did not. Stratified analyses were performed according to the American Joint Committee on Cancer (AJCC) stage. The Cox regression model was used to identify the risk factors for SPM and evaluate their effects.

**Results:**

We enrolled 85,947 eligible patients with BC, and 2,656 (3.09%) patients developed SPM. The median duration of SPM was 2.70 (1.14–5.14) years. Radiotherapy was administered in 40,946 (47.64%) patients, and chemotherapy was administered in 52,120 (60.64%). The most common SPMs were digestive tract cancers (876, 31.89%). The risk factors for SPM included the AJCC stage, chemotherapy, radiotherapy, age, and underlying comorbidities. Neither chemotherapy nor radiotherapy was associated with an increased risk of SPM in any stage. In contrast, after adjusting for other risk factors, patients at stage III/IV who received both therapies had lower risks of SPM compared with those who did not (*p* = 0.047).

**Conclusion:**

The risk of SPM was different across BC stages. Neither chemotherapy nor radiotherapy was associated with an increased risk of SPM in women with BC.

## Introduction

The incidence of breast cancer (BC) has gradually increased and is currently the leading one in women worldwide (1). This trend is observed in Western developed countries and Asia (2, 3). In addition to the increase in incidence, the survival of patients with BC has significantly improved in recent decades because of advances in medical care (4–6). In Taiwan, the 5-year survival rate for women diagnosed with BC in 1997 was 81% (7). As the prevalence and survival times of patients with BC have increased, so has research on long-term complications after anticancer therapies. One such late effect related to cancer treatment is the occurrence of second primary malignancies (SPMs), defined as the development of a new (not in the breast) cancer. Consequently, the prevalence and risk of SPM in patients with BC has become an important medical issue (8).

According to several epidemiological reports, women with BC have a higher risk of developing SPM than the general population, with a risk between 1.15 and 1.5 (9–12). Possible risk factors include younger age at diagnosis of BC, especially <40 years, and having received chemotherapy or radiotherapy. For BC treatment, in addition to surgical resection, the administration of other therapies, such as chemotherapy or radiotherapy, depends on the characteristics and stage of the cancer. Chemotherapy agents, such as alkylating agents and topoisomerase II inhibitors, have the potential to act as carcinogenic factors (13). Ionizing radiation is a carcinogen to humans, and radiotherapy may lead to the development of SPM (14–18), by damaging the irradiated cells directly or by affecting the cells not in the irradiated field through the bystander effects (14). However, few studies have evaluated the relationship between BC stages and SPM.

In this cohort study, we identified female patients with BC from the Breast Cancer Health Database (BCHD) of Taiwan. The BCHD includes national BC registration data with a stage definition by the American Joint Committee on Cancer (AJCC) stage system from the Taiwan Cancer Registry (TCR). We evaluated the incidence of SPM in different AJCC stages. We also compared the risk of SPM between women with BC who received chemotherapy and/or radiotherapy and those who did not. We expect this method to provide a more precise risk evaluation for SPM in women with different BC stages.

## Materials and methods

### Data source

The Health and Welfare Data Science Center (HWDC) set an integrated database to provide complete information on the Taiwanese National Health Insurance (NHI) reimbursement datasets, TCR, and death registration for the Taiwanese population. The NHI reimbursement datasets, which involved a single-payer insurance system, consisted of detailed healthcare information covering >99% of Taiwan’s total population, and included inpatient and ambulatory care claims from 1996 to 2015. The TCR included information on individual demographics, cancer stages, cancer primary sites, tumor histology, and treatment types. The data from the TCR included those collected through the Cancer Registry Annual Report Database, Taiwan Cancer Registry Long Form, and Taiwan Cancer Registry Short Form. The database from the HWDC was released with a de-identified format and was only used for research purposes. This study was conducted in compliance with the Declaration of Helsinki and was approved by the Research Ethics Committee of Chi Mei Hospital (IRB No. 10708-E04).

### Study subjects and definition

**Figure 1:**
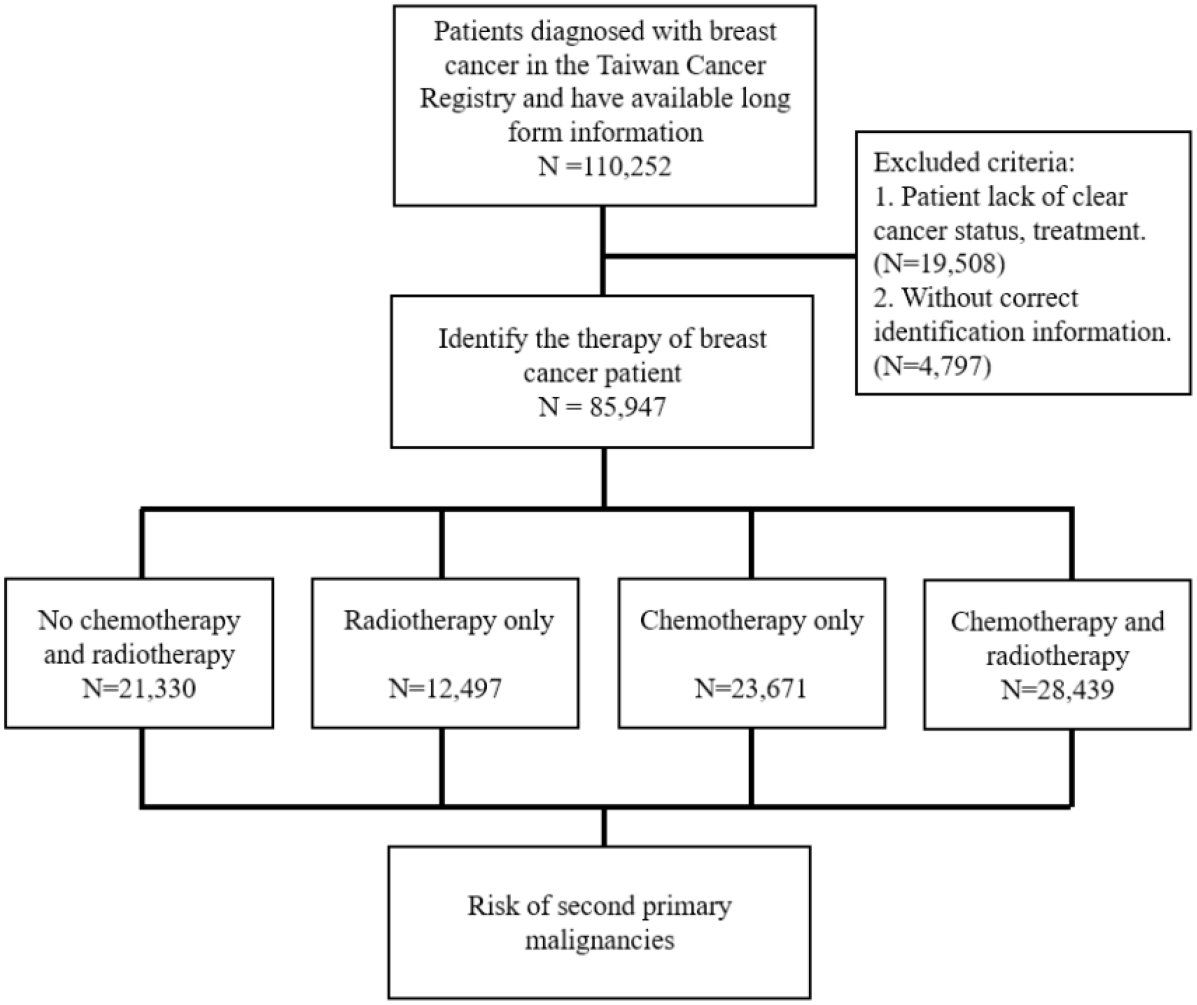
Diagram of risk evaluation for second primary malignancies from eligible women with breast cancer. ICD-9-CM: International Classification of Diseases, Ninth Revision, Clinical Modification, ICD-O-3: International Classification of Diseases for Oncology, Third Edition.

The study subjects, the patients with BC, were selected from the TCR from 2002 to 2014 in Taiwan based on the coding of the International Classification of Diseases, Ninth Revision, Clinical Modification (ICD-9-CM) and International Classification of Diseases for Oncology, Third Edition (ICD-O-3). Patients with ICD-9-CM code 174 were recognized as cases of BC. Patients with incomplete information in the TCR were excluded from this study. In addition, the age at diagnosis was categorized 18–39, 40–49, 50–59, and ≥60 years. Information on the chemotherapy and radiotherapy that the patient received and the comorbidities within 1-year before the diagnosis date was also collected. The comorbidities were identified using the ICD-9-CM, and the selected comorbidities included diabetes mellitus (DM; ICD-9-CM: 250), chronic obstructive pulmonary disease (COPD; ICD-9-CM: 490–496), liver diseases (ICD-9-CM: 571), and chronic kidney disease (CKD; ICD-9-CM: 585). The selected women with BC were grouped according to those who underwent chemotherapy and/or radiotherapy and were compared with women with BC who received neither therapy (**Figure 1**).

In this study, SPM was defined as a *de novo* new primary cancer diagnosed after the diagnosis of BC, which was not in the breast, nor synchronous or metachronous from BC. Specifically, it was identified using the ICD-9-CM and ICD-O-3 coded by the TCR. The second cancer types included head and neck (ICD-9-CM: 140–149; ICD-O-3: C00–C14); digestive (ICD-9-CM: 150–159; ICD-O-3: C15–C26 and C48); respiratory (ICD-9-CM: 160–165; ICD-O-3: C30–C39); bone, skin, and soft tissue (ICD-9-CM: 170–173); & (C40–C41, C44, C47, and C49); urinary (ICD-9-CM: 188–189; ICD-O-3: C64–C68); uterine (ICD-9-CM: 182; ICD-O-3: C54); ovarian (ICD-9-CM: 183; ICD-O-3: C56 and C570–C574); cervical (ICD-9-CM: 180; ICD-O-3: C53); hematological (ICD-9-CM: 200–208; ICD-O-3: C81–C96); and others (ICD-9-CM: 190–195 and 199; ICD-O-3: C69–C76 and C80). Synchronous and metachronous cancers (ICD-9-CM: 196–198; ICD-O-3: C77–C79) were excluded.

### Statistical analysis

Categorical variables were presented as frequency with percentage. The distribution differences between patients with and without SPM were evaluated using the Pearson’s chi-square test for categorical variables, including stage, therapy, age, differentiated grade, and comorbidities. In addition, the difference in follow-up time between patients with and without SPM was evaluated using the Wilcoxon rank-sum test after failing the normality test. The trends of changes across cancer stage were evaluated using the Cochran–Armitage test. To estimate the risk of SPM among patients with BC who received chemotherapy and/or radiotherapy, the Cox proportional regression was used to evaluate the potential variables. The trend of the cumulated incidence rate of SPM among patients with BC was plotted using the Kaplan–Meier approach, with the log-rank test to evaluate the difference. All analyses were conducted using the SAS statistical software Version 9.4 (for Windows; SAS Institute, Cary, NC, USA). The Kaplan–Meier curves were generated using Stata Version 12 (Stata Corp., College Station, TX, USA). The level of statistical significance was set at 0.05 (two-tailed).

## Results

### Baseline characteristics of patients with BC

We identified a total of 85,947 patients with BC in the BCHD. The distribution of patients with stage 0, I, II, and III/V were 11,149, 26,982, 35,458, and 12,358 respectively. The median age of BC diagnosis was 52 years. Most patients with BC were diagnosed between 40 and 69 years. In stages 0 to II, women aged 40–49 years constituted the highest proportion of patients with BC, but in stage III/IV, most patients were diagnosed at ≥60 years of age. “Moderately differentiated” was the most common grade of BC in all patients and in every stage division. DM was the most common among the comorbidities evaluated. The percentage of patients with DM gradually increased with the stage of BC, from 8.57% in stage 0 to 11.39% in stage III/IV. A total of 40,946 (47.64%) patients with BC had received radiotherapy, and the majority had stage I (14,213/26,982: 52.68%). Chemotherapy was performed in 52,120 (60.64%) patients with BC. The frequency of chemotherapy treatment increased with the stage (from 8.3%, 52.63%, 75.63%, to 82.36% in stage 0, I, II, to stage III/IV).

The median follow-up period was 4.25 years (interquartile range [IQR]: 1.96–6.13), with a total of 38, 0339.68 person-years. In total, 2,656 (3.09%) patients were documented with SPM. The mean time to develop SPM was 3.41 years (standard deviation [SD]: 2.81 years). The percentage of SPM gradually increased from stage 0 to II (2.51%, 3.30%, and 3.39% respectively) but declined to 2.29% in stage III/IV. Meanwhile, the time interval to develop SPM also increased from stage 0 to stage II but decreased in stage III/IV (2.78, 3.42, 3.74, and 2.58 years, respectively) (**Table 1**).

**Table 1:** Baseline characteristics of patients with breast cancer.

			Stage		
Characteristic	Total	0	I	II	III/IV
Number (%)	85947	11149	26982	35458	12358
No second primary malignancy	83291 (96.91)	10869 (97.49)	26092 (96.7)	34255 (96.6)	12075 (97.71)
Second primary malignancy	2656 (3.09)	280 (2.51)	890 (3.30)	1203 (3.39)	283 (2.29)
Time to second primary malignancy					
Mean follow-up, year (SD)	3.41 (2.81)	2.78 (2.41)	3.42 (2.76)	3.74 (2.89)	2.58 (2.68)
Median follow-up, year (IQR)	2.70 (1.14–5.14)	2.16 (0.80–4.05)	2.75 (1.19–5.10)	3.11 (1.35–5.64)	1.73 (0.39–3.75)
Follow-up person-year	380339.68	44853.70	128811.17	167366.21	39308.60
Median follow-up, year (IQR)	4.25 (1.96–6.13)	3.49 (1.60–5.77)	4.24 (1.96–7.13)	4.14 (1.93–7.03)	2.41 (1.07–4.58)
Median diagnosed age, year	52	51	52	52	54
Age (year) (%)					
18–39	8879 (10.33)	1000 (8.97)	2699 (10)	4054 (11.43)	1126 (9.11)
40–49	26502 (30.84)	3822 (34.28)	8786 (32.56)	10733 (30.27)	3161 (25.58)
50–59	26806 (31.19)	3737 (33.52)	8518 (31.57)	10606 (29.91)	3945 (31.92)
≥60	23760 (27.64)	2590 (23.23)	6979 (25.87)	10065 (28.39)	4126 (33.39)
Differentiated grade (%)					
Well	12607 (14.67)	2075 (18.61)	6096 (22.59)	3823 (10.78)	613 (4.96)
Moderately	39251 (45.67)	4439 (39.82)	13500 (50.03)	16473 (46.46)	4839 (39.16)
Poorly	24928 (29)	2777 (24.91)	5576 (20.67)	12488 (35.22)	4087 (33.07)
Undifferentiated	37 (0.04)	7 (0.06)	10 (0.04)	17 (0.05)	3 (0.02)
Not stated	9124 (10.62)	1851 (16.6)	1800 (6.67)	2657 (7.49)	2816 (22.79)
Comorbidities (%)					
DM	8817 (10.26)	955 (8.57)	2487 (9.22)	3967 (11.19)	1408 (11.39)
COPD	2841 (3.31)	370 (3.32)	890 (3.3)	1186 (3.34)	395 (3.2)
Liver	2159 (2.51)	292 (2.62)	795 (2.95)	864 (2.44)	208 (1.68)
CKD	1336 (1.55)	174 (1.56)	408 (1.51)	554 (1.56)	200 (1.62)
Radiotherapy (%)	40946 (47.64)	4179 (37.48)	14213 (52.68)	16484 (46.49)	6070 (49.12)
Chemotherapy (%)	52120 (60.64)	925 (8.3)	14200 (52.63)	26817 (75.63)	10178 (82.36)

AJCC, American Joint Committee on Cancer; CKD, chronic kidney disease; COPD, chronic obstructive pulmonary disease; DM, diabetes mellitus, IQR, interquartile range; SD, standard deviation.

### Second cancer types in patients with BC according to stage

Digestive tract cancers were the most common SPM, followed by respiratory tract cancers. When evaluating trends in cancer types from stage 0 to II, we found the risks of head and neck, digestive tract, bone/skin/soft tissue, and cervical cancers had significant increasing trends (all *p* < 0.05). However, none of the trends of the four cancer types were significant when evaluated from stage 0 to III/IV. Only the risk of respiratory tract cancers showed a significant decreasing trend (*p* = 0.010) (**Table 2**).

**Table 2:** Second cancer types in patients with different stages of breast cancer.

Cancer type (ICD-9 code) and (ICD-O-3 code)	Total			Stage			
		0	I	II	^a^ *p*-value	III/IV	^b^ *p*-value
Head and neck (140–149) and (C00–C14)	64 (0.07)	5 (0.04)	16 (0.06)	37 (0.10)	0.020	6 (0.05)	0.292
Digestive (150–159) and (C15–C26 and C48)	876 (1.02)	71 (0.64)	291 (1.08)	423 (1.19)	<0.001	91 (0.74)	0.190
Respiratory (160–165) and (C30–C39)	413 (0.48)	57 (0.51)	153 (0.57)	160 (0.45)	0.165	43 (0.35)	0.010
Bone, skin, and soft tissues (170–173) and (C40–C41, C44, C47, and C49)	124 (0.14)	11 (0.10)	36 (0.13)	68 (0.19)	0.014	9 (0.07)	0.667
Urinary (188–189) and (C64–C68)	186 (0.22)	19 (0.17)	76 (0.28)	73 (0.21)	0.871	18 (0.15)	0.184
Uterus (182) and (C54)	258 (0.30)	32 (0.29)	85 (0.31)	121 (0.34)	0.353	20 (0.16)	0.203
Ovarian (183) and (C56 and C570–C574)	90 (0.10)	9 (0.08)	30 (0.11)	43 (0.12)	0.292	8 (0.06)	0.874
Cervical (180) and (C53)	272 (0.32)	22 (0.20)	92 (0.34)	123 (0.35)	0.042	35 (0.28)	0.280
Hematological (200–208) and (C81–C96)	156 (0.18)	16 (0.14)	45 (0.17)	74 (0.21)	0.111	21 (0.17)	0.321
Others (190–195, 199) and (C69–C76 and C80)	308 (0.36)	44 (0.39)	100 (0.37)	124 (0.35)	0.469	40 (0.32)	0.314

The p-value was derived from the Cochran–Armitage trend test. ^a^Trend from stage 0 to II; ^b^trend in all stages.

ICD-9, International Classification of Diseases, Ninth Revision; ICD-O-3, International Classification of Diseases for Oncology, Third Edition.

### Overall risks for SPM

In the analysis for the risks of SPM, we defined patients with BC who did not receive chemotherapy nor radiotherapy as no therapy. The cancer stage, chemotherapy, radiotherapy, age, tumor differentiation grade, DM, COPD, liver diseases, and CKD were all significantly associated with the development of SPM (all *p* < 0.001) (**Table 3**). Specifically, compared with no therapy, chemotherapy was associated with a 25% less risk for SPM, radiotherapy was associated with a 10% less risk, and both therapies combined was associated with a 28% less risk.

**Table 3:** Overall demographic information of patients with breast cancer with and without second primary malignancy.

	Second primary malignancy		*p*-value*
	No N (%) = 83,291 (96.91)	Yes N (%) = 2,656 (3.09)	
Stage			
0	10869 (13.05)	280 (10.54)	<0.001
I	26092 (31.33)	890 (33.51)	
II	34255 (41.13)	1203 (45.29)	
III/IV	12075 (14.50)	283 (10.66)	
Therapy			<0.001
No	20549 (24.67)	781 (29.52)	
RT only	12156 (14.59)	341 (12.89)	
CH only	22892 (27.48)	779 (29.44)	
Both	27694 (33.25)	745 (28.16)	
Age (year)			<0.001
18–39	8748 (10.50)	131 (4.93)	
40–49	25899 (31.09)	603 (22.70)	
50–59	25972 (31.18)	834 (31.40)	
≥60	22672 (27.22)	1088 (40.96)	
Differentiated grade			0.005
Well	12208 (14.66)	399 (15.02)	
Moderately	37975 (45.59)	1276 (48.04)	
Poorly	24239 (29.10)	689 (25.94)	
Undifferentiated/not stated	8869 (10.65)	292 (10.99)	
Comorbidities			
DM	8422 (10.11)	395 (14.87)	<0.001
COPD	2720 (3.27)	121 (4.56)	<0.001
Liver	2019 (2.42)	140 (5.27)	<0.001
CKD	1364 (1.64)	72 (2.71)	<0.001
Median follow-up, year (IQR)	3.79 (1.72–6.63)	2.70 (1.14–5.14)	<0.001

*p-value was derived from Pearson’s Chi-square test for stage, therapy, age, differentiated grade, comorbidities, or Wilcoxon rank-sum test for follow-up time.

CH, chemotherapy; CKD, chronic kidney disease; COPD, chronic obstructive pulmonary disease; DM, diabetes mellitus; IQR, interquartile range; RT, radiotherapy.

### Risks for SPM according to BC stage

In the stratified analysis by AJCC stage, all the factors studied, including therapeutic strategies (radiotherapy only, chemotherapy only, both radiotherapy and chemotherapy, and neither), age, tumor differentiation grade, and all comorbidities, were related to SPM in stage I and II (**Tables 4A**, **B**). In contrast, only age and comorbidity of liver disease were related to SPM in stage 0 and III/IV (**Tables 4C**, **D**).

**Table 4a:** Risk of second primary malignancies in patients with stage I breast cancer.

	Second primary malignancy		*p*-value*
	No N (%) = 26092 (96.70)	Yes N (%) = 890 (3.30)	
Therapy			<0.001
No	6391 (24.49)	287 (32.25)	
RT only	5933 (22.74)	171 (19.21)	
CH only	5876 (22.52)	215 (24.16)	
Both	7892 (30.25)	217 (24.38)	
Age (years)			<0.001
18–39	2659 (10.19)	40 (4.49)	
40–49	8588 (32.91)	198 (22.25)	
50–59	8219 (31.50)	299 (33.60)	
≥60	6626 (25.39)	353 (39.66)	
Differentiated grade			<0.001
Well	5906 (22.64)	190 (21.35)	
Moderately	13049 (50.01)	451 (50.67)	
Poorly	5416 (20.76)	160 (17.98)	
Undifferentiated/not stated	1721 (6.60)	89 (3.30)	
Comorbidities			
DM	2359 (9.04)	128 (14.38)	<0.001
COPD	846 (3.24)	44 (4.94)	0.005
Liver	746 (2.86)	49 (5.51)	<0.001
CKD	479 (1.45)	29 (3.26)	<0.001
Median follow-up, year (IQR)	4.29 (2.00–7.21)	2.74 (1.19–5.06)	<0.001

*p-value was derived from Pearson’s Chi-square test for therapy, age, differentiated grade, comorbidities, or Wilcoxon’s rank-sum test for follow-up time.

CH, chemotherapy; CKD, chronic kidney disease; COPD, chronic obstructive pulmonary disease; DM, diabetes mellitus; IQR, interquartile range; RT, radiotherapy.

**Table 4b:** Risk of second primary malignancies in patients with stage II breast cancer.

	Second primary malignancy		*p*-value*
	No N (%) = 34255 (96.61)	Yes N (%) = 1203 (3.39)	
Therapy			<0.001
No	6195 (18.08)	282 (23.44)	
RT only	2091 (6.10)	73 (6.07)	
CH only	12045 (35.16)	452 (37.57)	
Both	13924 (40.65)	386 (2.77)	
Age (year)			<0.001
18–39	3986 (11.64)	68 (5.65)	
40–49	10470 (30.56)	263 (21.86)	
50–59	10266 (29.97)	340 (28.26)	
≥60	9533 (27.83)	532 (44.22)	
Differentiated grade			0.002
Well	3681 (10.75)	142 (11.80)	
Moderately	15875 (46.34)	598 (49.71)	
Poorly	12126 (35.40)	362 (30.09)	
Undifferentiated/not stated	2573 (7.51)	101 (8.40)	
Comorbidities			
DM	3769 (11.00)	198 (16.46)	<0.001
COPD	1131 (3.30)	55 (4.57)	0.016
Liver	800 (2.34)	64 (5.32)	<0.001
CKD	524 (1.53)	30 (2.49)	0.008
Median follow-up, year (IQR)	4.18 (1.96–7.10)	3.11 (1.35–5.65)	<0.001

*p-value was derived from Pearson’s chi-square test for therapy, age, differentiated grade, comorbidities, or Wilcoxon’s rank-sum test for follow-up time.

CH, chemotherapy; CKD, chronic kidney disease; COPD, chronic obstructive pulmonary disease; DM, diabetes mellitus; IQR, interquartile range; RT, radiotherapy.

**Table 4c:** Risk of second primary malignancies in patients with stage 0 breast cancer.

	Second primary malignancy		*p*-value*
	No N (%) = 10869 (97.49)	Yes N (%) = 280 (2.51)	
Therapy			0.666
No	6276 (57.74)	169 (60.36)	
RT only	3693 (33.98)	86 (30.71)	
CH only	512 (4.71)	13 (4.64)	
Both	388 (3.57)	12 (4.29)	
Age (year)			<0.001
18–39	992 (9.13)	8 (2.36)	
40–49	3748 (34.48)	74 (26.43)	
50–59	3631 (33.41)	106 (37.86)	
≥60	2498 (22.98)	92 (32.86)	
Differentiated grade			0.519
Well	2022 (18.60)	53 (18.93)	
Moderately	4330 (39.84)	109 (38.93)	
Poorly	2714 (24.97)	63 (22.50)	
Undifferentiated/not stated	1803 (16.59)	55 (19.64)	
Comorbidities			
DM	926 (8.52)	29 (10.36)	0.278
COPD	357 (3.28)	13 (4.64)	0.210
Liver	274 (2.52)	18 (6.43)	<0.001
CKD	168 (1.55)	6 (2.14)	0.426
Median follow-up, year (IQR)	3.52 (1.62–5.82)	2.16 (0.80–4.05)	<0.001

*p-value was derived from Pearson’s Chi-square test for therapy, age, differentiated grade, comorbidities, or Wilcoxon’s rank-sum test for follow-up time.

CH, chemotherapy; CKD, chronic kidney disease; COPD, chronic obstructive pulmonary disease; DM, diabetes mellitus; IQR, interquartile range; RT, radiotherapy.

**Table 4d:** Risk of second primary malignancies in patients with stage III/IV breast cancer.

	Second primary malignancy		*p*-value*
	No N (%) = 12075 (97.71)	Yes N (%) = 283 (2.29)	
Therapy			0.887
No	1687 (13.97)	43 (15.19)	
RT only	439 (3.64)	11 (3.89)	
CH only	4459 (36.93)	99 (34.98)	
Both	5490 (45.47)	130 (45.94)	
Age (year)			0.049
18–39	1111 (9.20)	15 (5.30)	
40–49	3093 (25.61)	68 (24.03)	
50–59	3856 (31.93)	89 (31.45)	
≥60	4015 (33.25)	111 (39.22)	
Differentiated grade			0.088
Well	599 (4.96)	14 (4.95)	
Moderately	4721 (39.10)	118 (41.70)	
Poorly	3983 (32.99)	104 (36.75)	
Undifferentiated/not stated	2772 (22.96)	47 (16.61)	
Comorbidities			
DM	1368 (11.33)	40 (14.13)	0.142
COPD	386 (3.20)	9 (3.18)	0.988
Liver	199 (1.65)	9 (3.18)	0.048
CKD	193 (1.60)	7 (2.47)	0.249
Median follow-up, year (IQR)	2.42 (1.08–4.59)	1.73 (0.39–3.75)	<0.001

*p-value was derived from Pearson’s chi-square test for therapy, age, differentiated grade, comorbidities, or Wilcoxon’s rank-sum test for follow-up time.

CH, chemotherapy; CKD, chronic kidney disease; COPD, chronic obstructive pulmonary disease; DM, diabetes mellitus; IQR, interquartile range; RT, radiotherapy.

### Hazard ratio of SPM in BC patients with radiotherapy and chemotherapy in different stages

**Figure 2:**
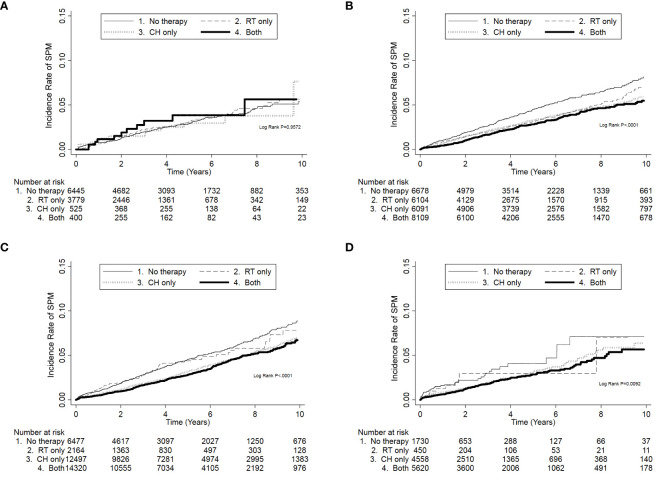
The cumulative incidence of developing second primary malignancies (SPMs) according to chemotherapy and radiotherapy in different American Joint Committee on Cancer breast cancer stages. **(A)** SPM risk was not associated with chemotherapy and radiotherapy in stage 0. **(B–D)** SPM was related to chemotherapy and radiotherapy in stages I, II, and III/IV. When both therapies were used, SPM risk was the lowest. CH, chemotherapy; RT, radiotherapy.

In the analysis of the associations of radiotherapy and chemotherapy with SPM in different stages, when patients without chemotherapy nor radiotherapy were used as the reference, neither chemotherapy nor radiotherapy were associated with an increased risk of SPM in any stage (**Table 5**; **Figure 2**). In contrast, patients who received chemotherapy and radiotherapy had a significantly lower risk of SPM in stages I, II, and III/IV (**Figures 2B–D**; *p* < 0.001 in stages I and II, *p* = 0.009 in stage III/IV). In the further analysis, patients who received radiotherapy only had a lower crude hazard ratio (HR) in stage I (crude HR = 0.76, *p* = 0.004). Meanwhile, patients who received chemotherapy only or both radiotherapy and chemotherapy had lower crude HRs in stages I, II, and III/IV. However, when adjusted for age, tumor differentiation grade, and comorbidities, only patients in stage III/IV who received both radiotherapy and chemotherapy had a lower risk of SPM (adjusted HR = 0.69, *p* = 0.047) (**Table 5**).

**Table 5:** Crude and adjusted hazard ratios of radiotherapy and chemotherapy for second primary malignancies in different stages.

	Crude HR (95% CI)	*p*-value	Adjusted HR* (95% CI)	*p*-value
Stage 0				
No	Ref.	–	Ref.	–
RT only	1.02 (0.79–1.32)	0.888	1.10 (0.84–1.43)	0.487
CH only	0.97 (0.55–1.70)	0.905	1.06 (0.60–1.88)	0.831
Both	1.28 (0.71–2.29)	0.413	1.46 (0.81–2.63)	0.210
Stage I				
No	Ref.	–	Ref.	–
RT only	0.76 (0.63–0.92)	0.004	0.90 (0.74–1.09)	0.284
CH only	0.73 (0.61–0.87)	<0.001	0.90 (0.75–1.09)	0.274
Both	0.64 (0.54–0.76)	<0.001	0.88 (0.73–1.07)	0.196
Stage II				
No	Ref.	–	Ref.	–
RT only	0.94 (0.73–1.22)	0.637	1.07 (0.83–1.39)	0.609
CH only	0.73 (0.63–0.85)	<0.001	0.85 (0.81–1.11)	0.498
Both	0.66 (0.57–0.77)	<0.001	0.95 (0.81–1.12)	0.539
Stage III/IV				
No	Ref.	–	Ref.	–
RT only	0.84 (0.43–1.63)	0.612	0.82 (0.42–1.59)	0.550
CH only	0.63 (0.44–0.91)	0.013	0.71 (0.49–1.02)	0.063
Both	0.61 (0.43–0.86)	0.005	0.69 (0.48–1.00)	0.047

*HR was adjusted for age, comorbidities, and differentiation grade.

CH, chemotherapy; HR, hazard ratio; RT, radiotherapy.

## Discussion

Few studies reported the risks of SPM at different stages of BC. A previous study used the Scandinavian Cancer Registration, which included Denmark, Finland, Norway, and Sweden, to evaluate second non-hematological malignancies of women with BC between 1943 and 2002. In that study, women with BC were divided into local, regional, and advance/distal status when diagnosed. The absolute risk of developing a second cancer was found to be dependent on stage and age; specifically, patients women aged <40 years and with localized disease had the greatest risk (11). However, some limitations of the study were noted. First, not all the countries had stage registration, and the starting year when the stage information became available was different across countries. Therefore, the definition and information of stages might not be coherent among countries. Second, the evaluated time interval was nearly 60 years with the earliest year dated back in 1943. Given the long study period, it is likely that the therapeutic strategies, medication, and treatment devices had large variations and improvements at different times and countries. Third, the impact of chemotherapy was not evaluated, and so the risk of developing SPM in patients at different BC stages remained unclear. In our study, the data were extracted from a single national Cancer Registration Databases with unified BC stage system in the recent decades, and the effects on SPM of chemotherapy and radiotherapy were evaluated. Our results were helpful to clarify this issue.

In our study, the incidence of SPM was 3.09% over a median follow-up of 4.25 years, which was similar to previous reports in Taiwan (12, 15). According to previous studies conducted in other areas, when increasing the follow-up time to 10 years, the incidence was reported as 4%–6% (11, 16). As the survival of patients with BC increases, the long-term follow-up, and monitoring of SPM become more important. Besides, we also found that the incidence of SPM gradually increased with higher AJCC stages from 0 to II; thus, the following strategies might be adapted for patients with BC in different AJCC stages.

In terms of the sites of SPM, digestive and respiratory tract cancers were the most common cancer types after BC. This may be due to the high incidence of both tract cancers in the general population (17). Besides, gynecological cancers, such as those of the uterus and cervix, and urinary tract and hematological cancers were also common after BC. However, when excluding patients with BC in stage III/IV, we found the risks of head and neck, digestive tract, skin/bone/soft tissue, and uterine cancers increased with an increase in the AJCC stage. The possible explanations for this may include the following: (1) Patients with BC may have underling hereditary genomic abnormities, resulting in a more invasive status at diagnosis and a higher risk of other malignancies (18). (2) The comorbidities of patients with BC might contribute to other cancers. Indeed, DM was more prominent in patients with higher BC stages, and several reports have found that DM was a potential risk factor for cancers, especially colorectal cancer. Moreover, a higher prevalence of DM was observed in patients with advanced BC (19, 20), and gastrointestinal tract cancers are the most commonly observed SPM. (3) As the treatment methods were different in different stages, the effects of therapy for BC in different stages might result in higher SPM risks in specific cancer sites. In our study, chemotherapy was performed more commonly in patients with higher stages of BC. Meanwhile, the percentage of radiotherapy was not coherent across different BC stages; thus, the diversities of radiotherapy and chemotherapy used in each stage might result in different second cancer sites. However, whether these factors relate to the types of SPM requires further evaluation.

Many factors were related to the occurrence of SPM. In our study, older patients with BC were more likely to have SPM than younger patients, especially those >60 years. This is likely because older patients have a greater likelihood of comorbidities such as DM, which are associated with the development of other cancers. However, the age at BC diagnosis was similar between patients in AJCC stages 0 and II; thus, the variation in SPM risk across different stages may not be solely due to age, and other factors should be investigated. All of the studied comorbidities were related to the occurrence of SPM, but the effect was not coherent across different AJCC stages. For example, in stage 0, only liver disease was related to SPM, but other diseases were not found to be significantly associated with SPM. Although some diseases might be considered to increase the risk of different types of cancer, their effects on the incidence of SPM after BC were unclear.

Chemotherapy and radiotherapy have the potential to act as carcinogenic factors (9); therefore, the increased risk of SPM after these therapies has become an important consideration for patients with BC. The causal relationships of leukemia and bladder cancer with chemotherapy had been reported (21, 22). For radiotherapy, previous (non-modern) radiotherapy delivery techniques have been clearly linked with SPM, especially the risk of BC in young female patients with Hodgkin lymphoma who were exposed to therapeutic radiation in thoracic-mediastinal fields. For young female with Hodgkin lymphoma, the late treatment-related SPM had been noticed and emphasized (23). A meta-analysis of randomized trials found that the average rate of SPM was approximately 1% per year for at least 30 years after treatment (24). A population-based study in 2002 evaluated the incidence of SPM in 32,591 patients with Hodgkin lymphoma by using data from National Cancer Institute’s Surveillance, Epidemiology, and End Results Program in the United States (25) and found that among female survivors, mantle field radiotherapy was associated with a drastically increased risk of secondary BC when compared with different fields of supradiaphragmatic irradiation (26–28). In addition, data from a nationwide cohort study in the United Kingdom with a 40-years follow-up showed that female patients with age range of 10–14 years had significant higher risk of SPM when compared with age range 30–35 years at the time of treatment (26). Likewise, a cohort study in the Netherlands also showed that the cumulative incidence of secondary BC was higher in patient treated at an age younger than 21 years (27). According to the previous studies, the association of radiotherapy and SPM depended on age and the field of radiation. In our studies, the use of chemotherapy led to a significantly lower risk of SPM in patients with AJCC stages I, II, and III/IV BC. Meanwhile, radiotherapy also led to a significantly lower SPM risk in stage I patients. Furthermore, patients with BC who received both chemotherapy and radiotherapy had a lower risk of SPM in AJCC stages I, II, and III/IV. After adjusting for other factors, the combination of both therapies was still associated with a lower SPM risk in patients with BC in stage III/IV. In higher AJCC stage, patients have a greater likelihood of receiving longer or different chemotherapy regimens, and the extended use of cytotoxic drugs may be associated with the risk of developing SPM. However, we did not find chemotherapy or radiotherapy to be associated with an increase in the risk of developing SPM. In fact, there were studies showing that adjuvant chemotherapy for BC was associated with a decreased risk of SPM (29, 30). Modern advances in radiotherapy delivery are also increasingly contributing to drastically change in reducing the risk of late sequelae in Hodgkin lymphoma (31, 32). According to our study results and recent epidemiological reports, we agree that patients with BC should receive optimal anticancer therapy, including chemotherapy and radiotherapy, as guided by the recommendation, and should not worry about SPM induced by chemotherapy or radiotherapy. However, further studies are required to confirm our findings.

Many epidemiological studies have reported a higher risk of SPM in women with BC than the general population, irrespective of whether the study population was European, American, or Asian (9–11, 33, 34). Several reasons have been proposed to explain the higher risk of SPM in women with BC, including women with BC might have underlying genetic abnormalities, such as *BRCA* gene mutation; shared similar environmental risk factors; therapeutic chemotherapy or radiotherapy; and more frequent surveillance after BC (9, 33). However, for patients with BC, especially those who were considered to be cured, it is important to recognize the possible factors leading to SPM as these factors help in preventing and monitoring SPM during the follow-up period. In our study, we evaluated SPM among women with BC themselves rather than comparing with the non-BC population. Therefore, the confounding biases of genetic diversities, environmental effects, and more frequent physical surveillance post-BC diagnosis were expected to be reduced in our study. Our results not only demonstrate that chemotherapy and radiotherapy have no clear association with SPM but also show that the underling comorbidities of patients with BC are important risk factors for SPM. We also evaluated the combined diseases of patients with BC and considered that controlling and monitoring these diseases is necessary in the prevention and early recognition of the occurrence of SPM.

Several limitations of our study should be recognized. First, we did not evaluate the hormone status and use of anti-hormone drugs. Endocrine therapy may influence the occurrence of SPMs; for example, the risk of endometrial cancer is increased in patients who received tamoxifen (35). However, the databases we used in this study did not have complete information on endocrine or target therapy, and so we were unable to evaluate their effects. Second, we did not have information on the genetic abnormalities of these patients, because the databases we used did not have information on genetic predisposition. Third, we have no information on the family history of cancer, physical status (e.g., body weight), daily habits (e.g., smoking and exercise), and occupational exposure of the patients. These factors may also be related to the occurrence of cancers. Forth, we could not determine the reasons why the patients received or did not receive chemotherapy or radiotherapy. Even in the same stages, some patients received chemotherapy, while others did not. These diversities might lead to different results in each stage.

In summary, our results showed that age, tumor differentiation grade, underlying comorbidities, cancer stages, chemotherapy, and radiotherapy were all associated with the occurrence of SPM in women with BC. We also found that these factors had different levels of influence for SPM across different BC stages. Further studies evaluating other factors and the interaction among those factors are necessary.

## Conclusions

Our results revealed that the risks of SPM varied across AJCC stages. In women with potential curative AJCC stages 0 to II BC, SPM risk gradually increased with higher stages. Meanwhile, chemotherapy or radiotherapy was not associated with an increased risk of developing SPM. We suggest that women with BC should receive chemotherapy and/or radiotherapy when clinically indicated. Further studies are warranted to evaluate other possible factors associated with SPM to provide a more comprehensive follow-up plan for female survivors of BC.

## Data availability statement

Publicly available datasets were analyzed in this study. This data can be found here: Health and Welfare Data Science Center of Taiwan.

## Ethics statement

The studies involving human participants were reviewed and approved by Research Ethics Committee of Chi Mei Medical Center. The ethics committee waived the requirement of written informed consent for participation.

## Author contributions

C-YL and H-RG conceived study. C-YL, S-YH, and H-RG designed the study. C-HH performed the data curation and statistical analysis. W-TH, C-JT, and S-BS helped interpret the data. The study was supervised by S-BS and H-RG. C-YL and S-YH wrote the manuscript, and others participated in the revision of the manuscript. All authors contributed to the article and approved the submitted version.
